# A Case of Transient, Isolated Cranial Nerve VI Palsy due to Skull Base Osteomyelitis

**DOI:** 10.1155/2014/369867

**Published:** 2014-06-15

**Authors:** Brijesh Patel, Anas Souqiyyeh, Ammar Ali

**Affiliations:** ^1^Department of Internal Medicine, Providence Hospital and Medical Center, 16001 W Nile Mile Road, Southfield, MI 48075, USA; ^2^Department of Infectious Disease, Providence Hospital and Medical Center, 16001 W Nile Mile Road, Southfield, MI 48075, USA

## Abstract

Otitis externa affects both children and adults. It is often treated with topical antibiotics, with good clinical outcomes. When a patient fails to respond to the treatment, otitis externa can progress to malignant otitis externa. The common symptoms of skull bone osteomyelitis include ear ache, facial pain, and cranial nerve palsies. However, an isolated cranial nerve is rare. Herein, we report a case of 54-year-old female who presented with left cranial nerve VI palsy due to skull base osteomyelitis which responded to antibiotic therapy.

## 1. Introduction 

Otitis externa, also known as swimmer's ear, is a commonly occurring disease. It affects the external ear structures causing pain (due to inflammation). When otitis externa fails to responds to therapy and progresses to affect the bony structures, malignant otitis externa (MOE) ensues. The infection from external ear to adjacent tissues and temporal bone spreads through the fissure of Santorini [1]. Skull base osteomyelitis (SBO) secondary to malignant otitis externa (MOE) was first described more than fifty years ago [2]. The clinical presentation of SBO included ear pain and discharge, sinusoidal pain, facial and periorbital swelling, and nasal stuffiness and discharge [3]. Other uncommon presentations of SBO also include cranial nerve palsies observed in 43.5% of patients involved mostly the facial nerve (VII) and combination with lower cranial nerves (CN VI, IX, X, XI, and XII) has also been described [4]. Isolated SBO cranial nerve deficits are rarely reported in literature [5, 6]. Herein, we report a case of transient abducens nerve palsy due to skull base osteomyelitis.

## 2. Case Report

**Figure 1:**
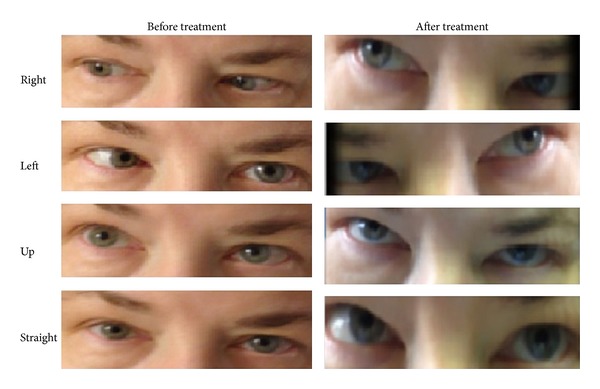
This image shows extraocular movements before and after the treatment.

A 54-year-old female presented to hospital for new onset of double vision. The patient denied having a similar episode in the past. Additionally, she had persistent left ear ache for 5 months. The patient stated that the earache started as dull, achy pain that had been progressively getting worse requiring multiple office visits. She had been treated with oral and topical antibiotics without any significant relief. Her past medical history includes diabetes mellitus, hypertension, obesity, hypothyroidism, and fibromyalgia. The patient's presenting vitals were stable. The ear exam revealed granulation tissue on the left tympanic membrane and serosanguinous fluid was present. The patient had painful left external ear structure upon gentle traction. The neurologic exam findings were significant for inability to abduct the left eye beyond midline (Figure 1). When looking left, the patient would have double vision. There were no other focal neurologic deficits, cranial nerve palsies, or abnormal cerebellar signs.

**Figure 2:**
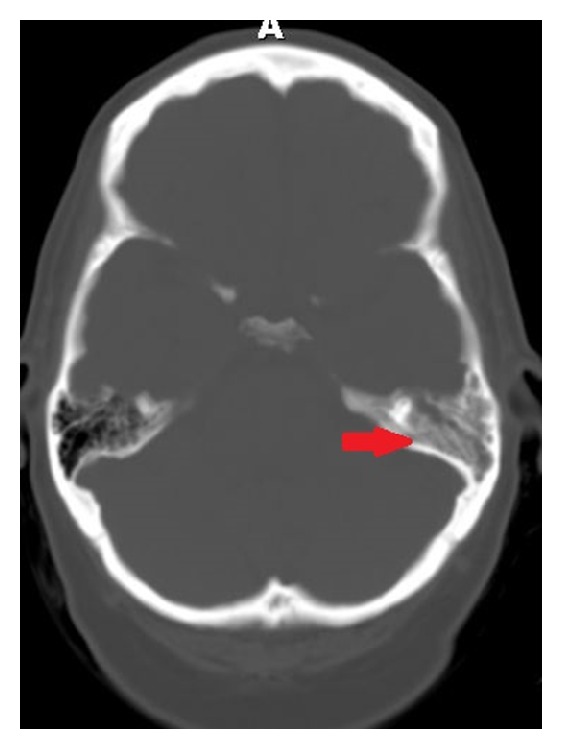
The CT scan shows nonspecific density in the temporal bone without bony erosion (red arrow).

**Figure 3:**
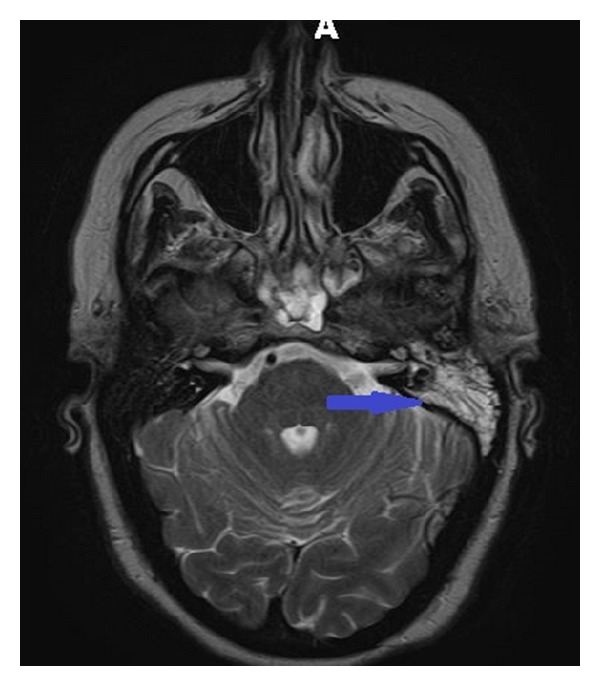
The MRI shows enhancement in the temporal bone region equivocal for inflammation and infection (blue arrow).

**Figure 4:**
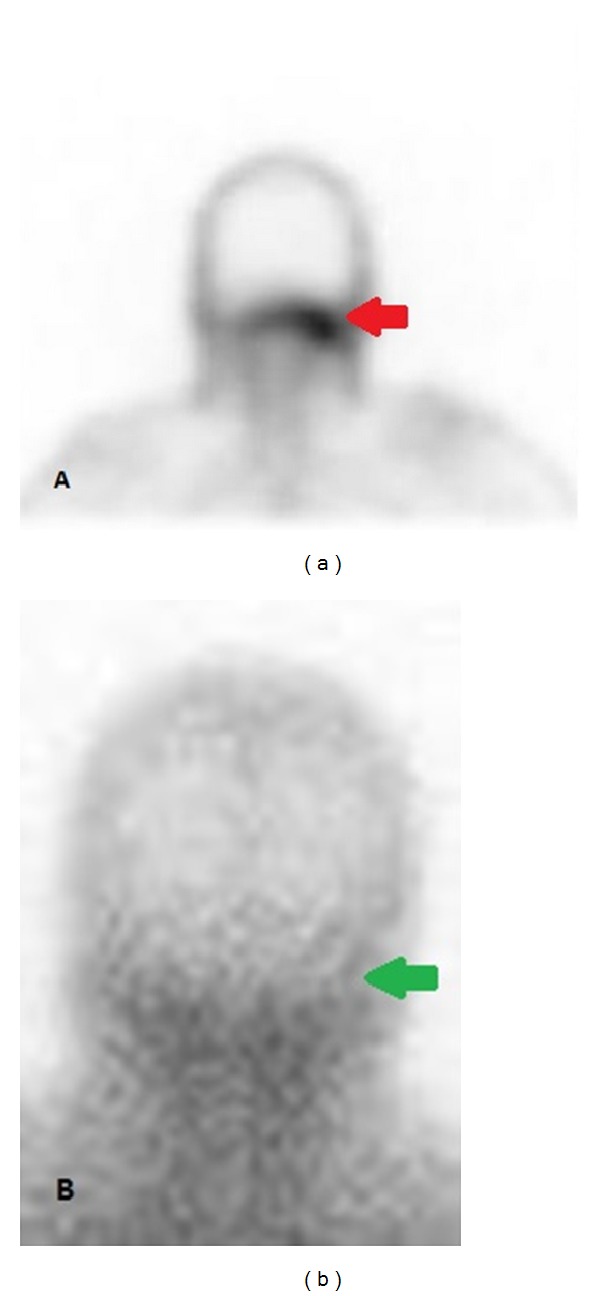
(a) The Gallium-67 scan before the treatment confirms the diagnosis of left SBO (red arrow). (b) The repeated Gallium-67 scan shows the resolution of the left SBO (green arrow).

A CT scan of the head was inconclusive (Figure 2). A magnetic resonance image (MRI) of the brain showed an enhancement of left skull base that raised the suspicion for active infection (Figure 3). These findings were further assessed with Gallium-67 citrate (Gallium-67 scan), which revealed a strong possibility of active infection (Figure 4(a)). Based on imaging and clinical findings, the patient was diagnosed with temporal bone osteomyelitis (TBO) due to malignant otitis externa. She was empirically treated with cefepime for possible* Pseudomonas* species infection. The patient underwent bone biopsy, which did not grow any organisms. The patient was treated with IV cefepime for 6 weeks. After the completion of antibiotic therapy, a repeat Gallium-67 scan showed improvement in the inflammation (Figure 4(b)). The patient was then treated with oral ciprofloxacin. The Gallium-67 scan five months after the initial scan showed complete resolution of the inflammation. Her diplopia has markedly improved (Figure 1). The patient follows up with an ophthalmologist and complete resolution of diplopia is expected.

## 3. Discussion

The most common organism that causes otitis externa or malignant otitis externa is* Pseudomonas aeruginosa* [7] since it tends to colonize in moist environment. Other microorganisms such as* Staphylococcus* species, and certain fungal infections can also cause MOE [8, 9]. Serious manifestation or involvement of MOE occurs in elderly patients, diabetics, and immunocompromised patients [1]. Once MOE involves the temporal bone, cranial nerves are susceptible to damage. The differential diagnosis of diplopia in this patient encompasses many ocular neuropathic etiologies. The three ocular nerves (III, IV, and VI) have been associated with diplopia as these nerves innervate the extraocular muscle. The implication of the MOE and SBO on affecting the cranial nerve is attributed to the pathway in which the nerve travels from the brain stem to the lateral rectus. The abducens nerve leaves the horizontal sulcus in the brain stem to enter the subarachnoid space over the petrous apex of the temporal bone at Dorello's canal and enters the cavernous sinus before finally entering the orbit [10]. Thus, the infection of temporal bone could affect the CN VI.

Once SBO is suspected, the best initial assessment modality of soft tissue was found to be an MRI [11]. A CT scan is very useful initial modality but fails to show the infection early. Nuclear scans used in SBO include gallium, Indium-111-labeled leukocyte scintigraphy (WBC Scan), technetium bone scan, and single photon emission computed tomography (SPECT) [12]. It is beneficial to use Gallium-67 scan for diagnosis and followup in MOE and SBO. The treatment directed against the infectious agent should be started over the span of at least 4 weeks [1, 13]. The choice of antibiotics should be guided by tissue culture and sensitivity [13]; however, in our case the biopsy culture was negative for any organism. She was treated for presumed* Pseudomonas aeruginosa*. After both intravenous and oral antibiotics, the patient has responded to the therapy with gradual recovery.

## 4. Conclusion

MOE/SBO is a rare cause of isolated CN VI palsy, which could be reversed with the successful treatment.
